# Targeted Exome Sequencing of Congenital Cataracts Related Genes: Broadening the Mutation Spectrum and Genotype–Phenotype Correlations in 27 Chinese Han Families

**DOI:** 10.1038/s41598-017-01182-9

**Published:** 2017-04-27

**Authors:** Yi Zhai, Jinyu Li, Wangshu Yu, Sha Zhu, Yinhui Yu, Menghan Wu, Guizhen Sun, Xiaohua Gong, Ke Yao

**Affiliations:** 1 Eye Center, Second Affiliated Hospital of Medical College, Zhejiang University, Hangzhou, Zhejiang China; 2 Key Laboratory of Ophthalmology of Zhejiang Province, Hangzhou, China; 3 The 117th hospital of PLA, Hangzhou, China; 4 Naval Convalescent Zone of Hangzhou Sanatorium, Nanjing Military Command, CPLA, Hangzhou, China; 5 School of Optometry and Vision Science Program, University of California, Berkeley, California United States of America

## Abstract

Congenital cataract is the most frequent inherited ocular disorder and the most leading cause of lifelong visual loss. The screening of pathogenic mutations can be very challenging in some cases, for congenital cataracts are clinically and genetically heterogeneous diseases. The aim of this study is to investigate the mutation spectrum and frequency of 54 cartaract-associated genes in 27 Chinese families with congenital cataracts. Variants in 54 cataract-associated genes were screened by targeted next-generation sequencing (NGS) and then validated by Sanger sequencing. We identified pathogenic variants in 62.96% (17/27) of families, and over 52.94% (9/17) of these variants were novel. Among them, three are splicing site mutations, four are nonsense mutations, seven are missense mutations, two are frame shift mutations and one is intronic mutation. This included identification of: complex ocular phenotypes due to two novel PAX6 mutations; progressive cortical cataract and lamellar cataract with lens subluxation due to two novel CRYGS mutations. Mutations were also found in rarely reported genes including CRYBA4, CRYBA2, BFSP1, VIM, HSF4, and EZR. Our study expands the mutation spectrum and frequency of genes responsible for congenital cataracts. Targeted next-generation sequencing in inherited congenital cataract patients provided significant diagnostic information.

## Introduction

Congenital cataract is the most frequent eye disease and the most leading cause of blindness in childhood, affecting tens of millions of people^1, 2^. The prevalence of congenital cataracts is approximately 1 to 6 per 10,000 live births, while 27–39% of which are believed to be inherited^3^. There are autosomal-dominant, autosomal-recessive, and X-linked genetic forms of congenital cataracts, which may be isolated or associated with other ophthalmic abnormalities and syndromic associations^4^.

So far, more than 40 genes have been reported to be associated with congenital cataracts (Cat-Map; http://cat-map.wustl.edu/)^5^. These genes code for a variety of lens proteins with structural and chaperone functions, including α-, β-, and γ-crystallins, lens-specific transmembrane gap junction protein genes (GJA3 and GJA8), membrane protein genes (MIP and LIM2), and lens-associated transcription factors (e.g. HSF4, PITX3, MAF, PAX6, and FOXE3). Structural proteins such as the lens-specific beaded filament protein genes (BFSP1 and BFSP2) represent an additional group of proteins that may have mutations leading to cataract formation^6^. For most of these genes, cataract is the only disease phenotype observed^7^.

In order to identify the genetic cause of our newly recruited 27 families with congenital cataracts, we applied targeted exome sequencing using SureSelect Target Enrichment Kit. 17 mutations were identified in the 27 families, and 13 mutations were considered to be novel. Mutations were identified in 12 genes and we found a high mutation detection rate of approximately 62.96% in these families.

## Results

### Next Generation Sequencing

The present study recruited 27 families with congenital cataract. Targeted exome sequencing results of the 27 probands detected 6,024 variants in the 54 known genes (Table S1). Bioinformatics analysis of these mutations revealed that 30 of them are potential pathogenic (Table S2).

### Validation by Sanger sequencing

**Figure 1:**
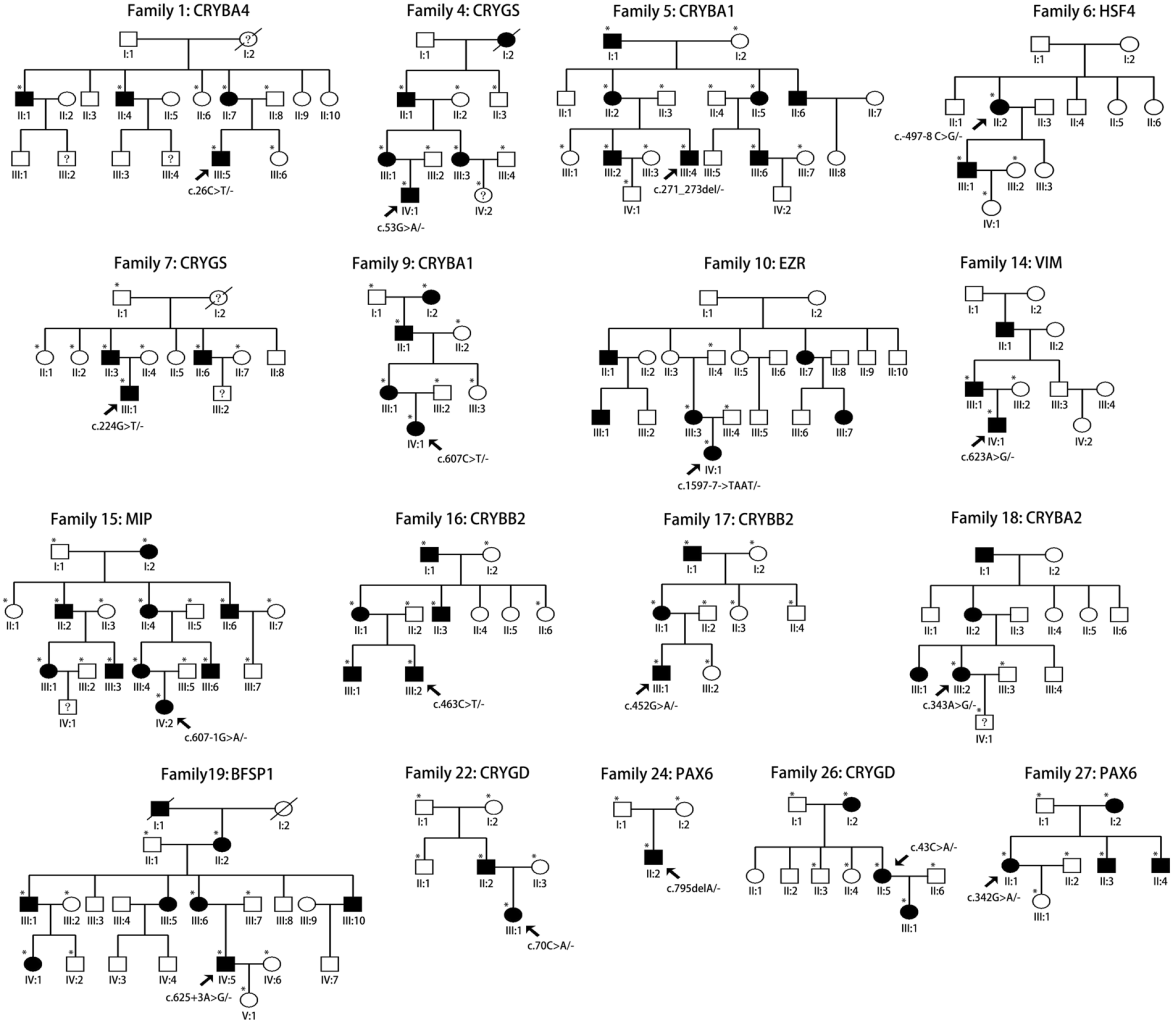
Pedigrees of the families with mutations. Squares indicate men and circles women; black and white symbols represent affected and unaffected individuals, respectively. The proband is marked with an arrow, and asterisks indicate those members enrolled in this study.

All of 30 mutations are confirmed by Sanger sequencing in probands and available family members. Among them, seventeen mutations were confirmed to be cosegregated with congenital cataracts (Table 1). SIFT predicts substitutions with scores less than 0.05 as deleterious, Polyphen-2 predicts substitutions with scores greater than 0.75 as “probably damaging”. The pedigrees of seventeen families are presented in Fig. 1.

**Table 1:** The pathogenic mutations identified in Chinese families with congenital cataract.

Family ID	Gene	Nucleotide	Amino acid	Mutation type	Status	Bioinformation prediction		Variant in controls	Note
						SIFT	Polyphen-2		
1	CRYBA4	c.26C > T	p.A9V	missense	Hetero	0.66	0	0/100	Novel
4	CRYGS	c.53G > A	p.G18D	missense	Hetero	0	0.989	0/100	Novel
5	CRYBA1	c.271_273delGGA	p.G91del	flame shift	Hetero	/	/	0/100	Novel
6	HSF4	c.-497-8C > G		intronic	Hetero	/	/	0/100	Novel
7	CRYGS	c.224_225GC > TT	p.G75V	missense	Hetero	0	0.999	0/100	Novel
9	CRYBA1	c.607C > T	p.Q203X	nonsense	Hetero	1	0.735289	0/100	Novel
10	EZR	c.1597-7insTAAT		splicing site	Hetero	/	/	0/100	Novel
14	VIM	c.623A > G	p.Q208R	missense	Hetero	0.07	0.712	0/100	Novel
15	MIP	c.607-1G > A		splicing site	Hetero	/	/	0/100	[8]
16	CRYBB2	c.463C > T	p.Q155X	nonsense	Hetero	0.01	0.641104	0/100	[9]
17	CRYBB2	c.452G > A	p.W151X	nonsense	Hetero	0	0.641681	0/100	Novel
18	CRYBA2	c.343A > G	p.N115D	missense	Hetero	0.22	0.004	0/100	Novel
19	BFSP1	c.625 + 3A > G		splicing site	Hetero	/	/	0/100	Novel
22	CRYGD	c.70C > A	p.P24T	missense	Hetero	0.05	0.102	0/100	[10]
24	PAX6	c.795delA	p.E265fs	flame shift	Hetero	/	/	0/100	Novel
26	CRYGD	c.43C > A	p.R15S	missense	Hetero	0	0.974	0/100	[11]
27	PAX6	c.342G > A	p.W114X	nonsense	Hetero	0	0.735284	0/100	Novel

**Figure 2:**
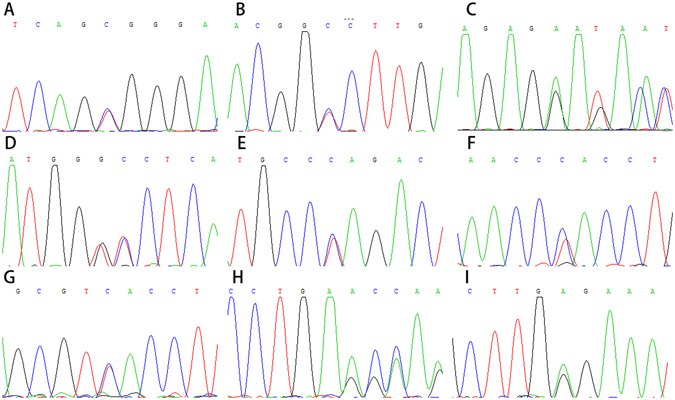
Sequencing results of nine novel disease-causing mutations. (**A**) Forward sequencing showed c.26C > T mutation of CRYBA4 gene in patients from family 1. (**B**) Reverse sequencing showed c.53G > A mutation of CRYGS gene in patients from family 4. (**C**) Forward sequencing showed p.G91del mutation of CRYBA1 gene in patients from family 5. (**D**) Forward sequencing showed c.224_225GC > TT mutation of CRYGS gene in patients from family 7. (**E**) Forward sequencing showed c.607C > T mutation CRYBA1 gene in patients from family 9. (**F**) Reverse sequencing showed c.452G > A of CRYBB2 gene in patientsfamily 17. (**G**) Reverse sequencing showed c.625 + 3A > G mutation of BFSP1gene in patients from family 19. (**H**) Forward sequencing showed c.795delA mutation of PAX6 gene in patient from family 24. (**I**) Forward sequencing showed c.342G > A mutation of PAX6gene in patients family 27.

Ten of seventeen mutations were identified in crystallin genes, while other seven mutations identified in six genes. Two mutations are in PAX6 (MIM: 607108); three mutations are in cytoskeletal protein (BFSP1 (MIM: 611391), VIM (MIM: 116300), and EZR); and one mutation each in MIP (MIM: 154050) and HSF4 (MIM: 602438). Among seventeen mutations, three are splicing site mutations, four are nonsense mutations, seven are missense mutations, two are frame shift mutations and one is intronic mutation. None of these seventeen mutations was detected in 100 controls. Nine mutations were considered as novel disease-causing mutations (DNA sequencing results provided in Fig. 2); while four have been previously linked to congenital cataracts^8–11^ (Figure S1). However, four pathogenic mutations (in family 6, 10, 14 and 18) could not be strongly associated with congenital cataracts due to the limited DNA samples of the family members and bioinformation prediction results (Figure S2).

### Clinical findings

**Figure 3:**
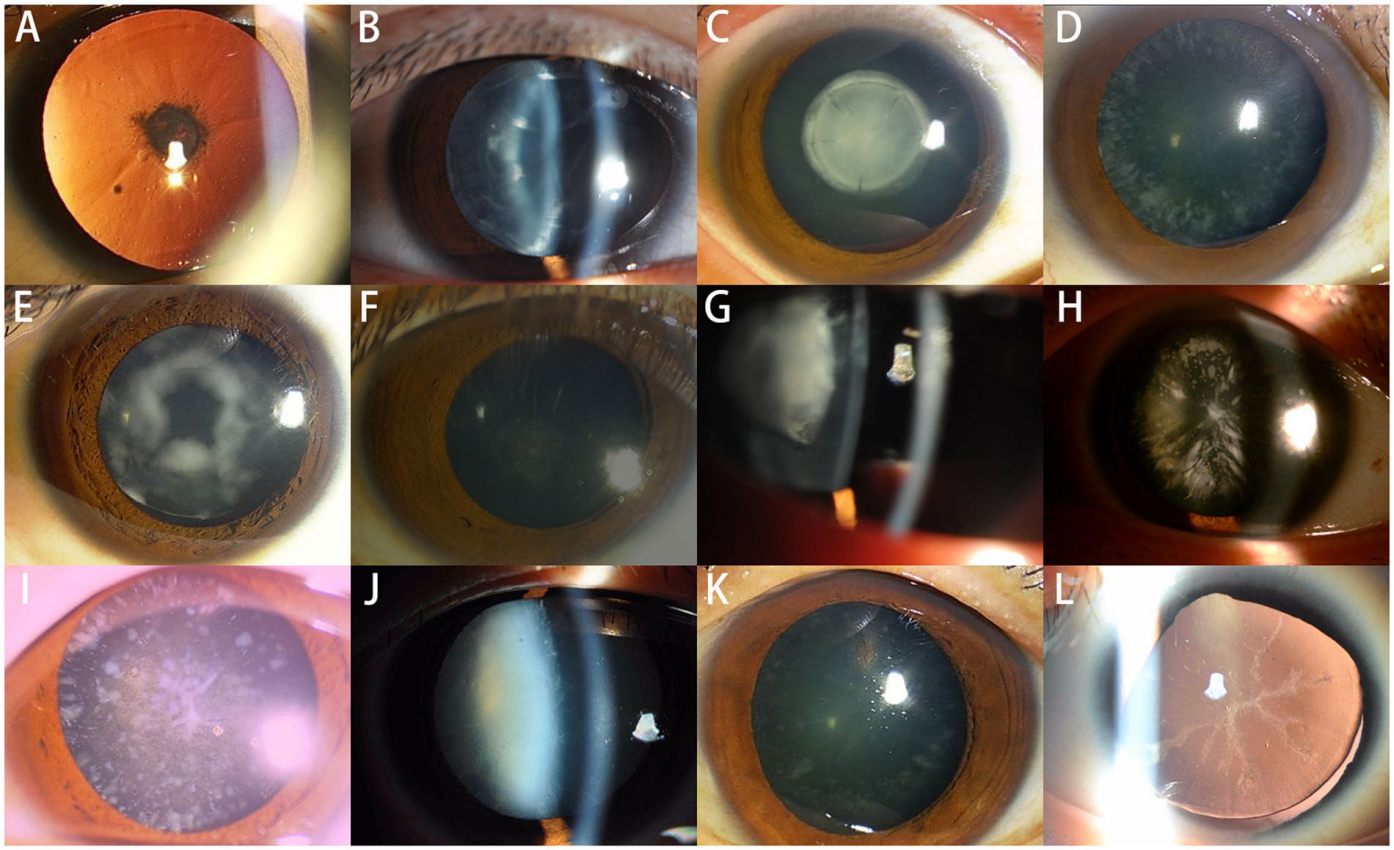
Phenotypes of the probands. (**A**) Photograph of proband in family 1 presented an anterior polar cataract. (**B**) Slit-lamp photograph of proband in family 4 showed a progressive cortical and sutural cataract. (**C**) Photograph of proband in family 5 showed a perinuclear zonular cataract. (**D**) Photograph of proband of family 6 showed a lamellar cataract with fine punctate opacities involving the cortical area of lens. (**E**) Photograph of proband in family 7 showed a subluxation of lens with a lamellar cataract. (**F**) Photograph of proband in family 14 presented a posterior polar cataract. (**G**) Slit-lamp photograph of proband in family 15 presented a nuclear cataract. (**H**) Photograph of proband in family 16 presented a cerulean cataract. (**I**) Photograph of proband in family 17 presented a cerulean cataract. (**J**) Slit-lamp photograph of proband in family 18 presented a total cataract. (**K**) Photograph of proband in family 19 showed a lamellar punctate cataract. (**L**) Photograph of proband in family 24 showed a coralliform cataract with aniridia.

All patients in this study had different types of congenital cataracts without other systemic diseases. Other ophthalmic findings of seventeen probands were listed in Table 2. Two families (family 24 and 27) with PAX6 mutation showed aniridia. Twelve phenotypes of probands with congenital cataract were recorded (Fig. 3), while other five probands underwent cataract surgery prior to this study. The phenotype of these families could only determinate by their medical record.

**Table 2:** Clinical features of affected probands with variants identified in this study.

Family ID	Variation	Sex	Age at examination (yrs)	Cataract types	Other clinical finding
Family 1	CRYBA4, c.26C > T	M	38	Anterior polar cataract	
Family 4	CRYGS, c.53G > A	M	7	Cortical and sutural cataract	Progressive
Family 5	CRYBA1, c.271_273delGAG	M	35	Zonular Cataracts	
Family 6	HSF4, c.-497-8 C > G	F	59	Lamellar, punctate	
Family 7	CRYGS, c.224_225GC > TT	M	8	Lamellar cataract	Lens subluxation
Family 9	CRYBA1, c.607C > T	F	3	Nuclear cataract	Nystagmus
Family 10	EZR, c.1597-7- > TAAT	F	3	Total cataract	Nystagmus
Family 14	VIM, c.623A > G	M	6	Posterior polar cataract	
Family 15	MIP, c.607-1G > A	F	1	Nuclear cataract	Nystagmus
Family 16	CRYBB2, c.463C > T	M	6	Cerulean cataract	
Family 17	CRYBB2, c.452G > A	M	2	Cerulean cataract	
Family 18	CRYBA2, c.343A > G	F	26	Total cataract	Progressive
Family19	BFSP1, c.625 + 3A > G	M	23	Lamellar, punctate	Progressive
Family 22	CRYGD, c.70C > A	F	2	Coralliform cataract	Nystagmus
Family 24	PAX6, c.795delA	M	7	Coralliform cataract	Nystagmus, aniridia
Family 26	CRYGD, c.43C > A	F	28	Coralliform cataract	Nystagmus
Family 27	PAX6, c.342G > A	F	28	Anterior and posterior polar cataract	Nystagmus, aniridia

## Discussion

More than 40 genes have been associated with congenital cataracts. Screening of these genes in groups of congenital cataract patients showed that the mutation frequencies have great differences^12–15^. Hansen *et al*. recruited 28 Danish families with hereditary congenital cataracts, and screened 17 cataract-related genes. He found that mutations in genes encoding crystallins and connexins account for 53.5% of inherited cataracts^14^. Dave *et al*. believed that EPHA2 mutations are major contributors to inherited cataracts in South-Eastern Australia^12^. Sun *et al*. indicated that mutations in NHS are the common causes of nonsyndromic congenital cataracts and account for 11.8% of the congenital cataracts^15^. In this study, we performed targeted exome sequencing on probands from 27 families with congenital cataracts. Sequence results indicated that 30 mutations are potentially pathogenic. Sanger sequencing confirmed that seventeen mutations are disease-causing. Our study revealed that mutations in crystallin genes are still the leading causes of nonsyndromic congenital cataracts with a frequency of 37.03%.

### Mutations in the Lens-Specific Crystallin Genes

Ten crystallins gene mutations were found in 27 families corresponding to 37.03% of the analyzed families, which is in the same magnitude as the percentage of crystallin mutations in Denmark group (36%)^14^. However, only 2 crystallin mutations (5%) were identified among 32 families with autosomal dominant congenital cataracts (ADCC) in southeastern Australia^16^. This difference of results may be influenced by different ethnic background and selection bias of family samples.

Three crystallins gene mutations have been associated with congenital cataracts. CRYGD p.P24T is a hotspot for mutation which has been reported for several times^10, 16–19^. Previous studies have showed different phenotypes (e.g. coralliform, cerulean, lamellar) of CRYGD p.P24T. Our proband showed a coralliform cataract, which is one of the most common phenotype of this mutation^20–23^. CRYBB2 p.Q155X is another hotspot for mutation in congenital cataracts^13, 24, 25^. Phenotypes of this mutation have been described as cerulean cataracts, which is also in correspondence with the proband of family16. CRYGD p.R15S has been reported once by Zhang and colleagues with a phenotype of coraliform cataracts^11^. The proband of family also present a coralliform cataract. Our results confirmed these recurrent mutations, and further expanded the mutation spectrum of congenital cataracts.

Two novel nonsense mutations CRYBB2 p.W151X and CRYBA1 p.Q203X may terminate the reading frame before the authentic stop codon. Nonsense-mediated decay (NMD) is the process by which mRNAs containing pre-mature termination codons (PTCs) are degraded before production of supposed truncated proteins^26, 27^. Two CRYGS mutations p.G18D and p.G75V has been detected in two families. The CRYGS p.G18V mutation has been associated with dominant progressive cortical cataract^28^, and reported to increase the gammaS-crystallin sensitivity to thermal and chemical stress^29^.

**Figure 4:**
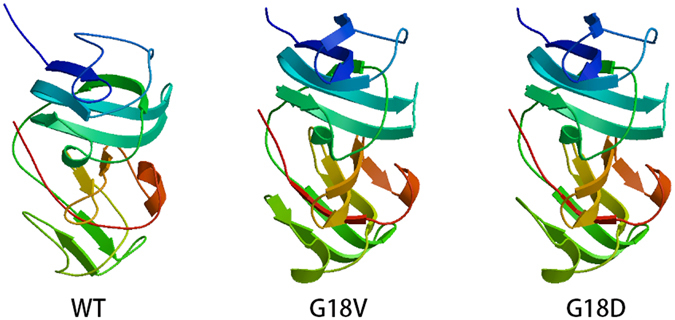
Stuctural modeling of WT, p.G18V and p.G18D crystallin gamma S using SWISS-MODEL.

Kingsley *et al*. suggested that the potential mechanism for CRYGS p.G18V mutation to cause cataract formation is the depletion of the finite αB-crystallin population of the lens^30^. The results of their study indicated normal association and structural properties of the G18V mutant γS-crystallin under mild conditions, but increased sensitivity stress, which were thus consistent with the progressive nature of the cataracts in the family. The CRYGS p.G18D mutation, located in the same locus of p.G18V, may also alter the sensitivity to thermal and chemical stress, and deplete αB-crystallin of the lens as well. SWISS-MODEL revealed both p.G18V and p.G18D are significantly different from wild type (Fig. 4). The phenotype of CRYGS p.G18D mutation is also progressive cortical and sutural cataract, and this is in accordance with the phenotype p.G18D caused.

The novel deletion mutation (c.271_273delGAG) in exon 4 of CRYBA1 was identified in a family with autosomal dominant congenital cataracts. Several deletion mutations have been identified in CRYBA1 gene^31, 32^ and CRYBA1c.272_274delGAG has been widely reported^33–36^. Xu indicated that DeltaG91 mutation of CRYBA1altered protein-protein interaction between human lens betaA1-crystallins, and lead to protein insolubilization and contribute to cataracts^37^. In our study, a novel in-frame deletion of three bp was dcted in exon 4 of CRYBA1 (c.271_273delGAG). Though this is a novel mutation on DNA level, it also leads to a DeltaG91 deletion like c.272_274delGAG mutation dose. Thus, this mutation was predicted to cause the same protein insolubilization of betaA1-crystallins as c.271_273delGAG dose.

The mutation found in CRYBA4 (c.26C > T, p.A9V) is the first cataract-associated CRYBA4 mutation with a dominant pattern. This mutation has been previously detected by Sun *et al*.^15^. They suggested that CRYBA4 p.A9V may be the pathogenic mutation of a Chinese family with congenital cataracts. But they cannot be sure due to bioinformation prediction results and limited family members. Our results confirmed that this mutation is cosegregated with congenital cataracts within the family, verified their hypothesis.

### Mutations in the cytoskeletal protein

The structural framework of lens cells is determined by the interaction of the cytoskeleton and the crystallins within the cytoplasm. Beaded filament is a type of intermediate filament which is unique to the lens fiber cells^6^. They are made up of BFSP1 (also called CP115 or filensin) and BFSP2 (also called CP49 or phakinin), highly divergent intermediate filament proteins that combine in the presence of crystallin to form the appropriate beaded structure^4^. Several different mutations of BFSP2 have been linked to ADCCs^38–40^, while BFSP1gene mutations have been linked to both autosomal dominant pattern (p.D348N)^41^ and autosomal recessive pattern (p.T246del74fsX6)^42^. To date, only these two BFSP1 disease-causing mutations have been reported. Thus, BFSP1 c.625 + 3A > G mutation we detected was the first report of BFSP1 splicing site mutation.

We also detected two cytoskeletal protein mutations EZR c.1597-7insTAAT and VIM p.Q208R. Lin *et al*. has linked several EZR mutations to age-related cataracts^43^. The mutation of VIM (p.E151K) is associated with inherited congenital cataracts. The mutant formed an aberrant vimentin cytoskeleton and increased the proteasome activity in transfected cells^44^. Thus, further investigation of EZR c.1597-7insTAAT and VIM p.Q208R are needed to clarify the pathogenicity of these two mutations.

### Mutations in PAX6 gene

Congenital aniridia with cataract is linked to a mutation of the PAX6 genes. Human PAX6 is composed of two DNA-binding domains: the paired domain (PD) of 128 amino acids and the homeodomain (HD) of 61 amino acids separated by a linker region of 79 amino acids, and is followed by a proline, serine, threonine-rich (PST) domain of 79 amino acids which have transcriptional trans-activation function^45^. It is a highly conserved transcription factor which regulates the tissue-specific expression of various molecules, hormones, and structural proteins. It is required for the development of the nervous system, eyes, nose, pancreas, and pituitary gland^46–48^.

As a crucial transcriptional factor, PAX6 mutations may affect a broad range of structures during development. Therefore, the phenotypes of different PAX6 mutations can be very diverse. PAX6 mutations is characterized by partial or complete absence of the iris accompanied with other ocular abnormalities such as cataract, glaucoma^49^, corneal degeneration^50^, microphthalmia^51^, foveal hypoplasia^52^, optic-nerve malformations^53^. Some individuals with PAX6 mutation developed other systemic diseases such hepatoblastoma, polydactylia^54^. PAX6 regulates numerous downstream genes, and its expression level is also regulated by several factors during eye development. Thus, the aniridia phenotype may vary even within the family, and the obvious genotype–phenotype correlation was very hard to identified^54^. However, Lin *et al*. reviewed the mutations archived in the PAX6 AllelicVariant Database, and found that over three-quarters of aniridia cases are caused by mutations that introduce a PTC into the open reading frame of PAX6^50^. It was widely belived that truncations of Pax6 can usually cause aniridia phenotype, due to haploinsufficiency^55^. Patients with PAX6 contiguous deletion, may have relatively severe phenotype, including bilateral complete absence of iris and foveal hypoplasia^49^. The two novel PAX6 mutations detected in our study were p.E265fs and p.W114X. Patient with p.E265fs mutation showed a partial absence of the iris, congenital coralliform cataracts and nystagmus (Fig. 3). This frameshift mutation is very close to p. E265fs. All patients in family 27 with p.W114X mutation showed a complete absence of iris, congenital anterior and posterior polar cataracts, as well as nystagmus. PAX6 nonsense mutations been widely reported (p.Arg240X, p.W100X, p.R103X, etc.), and linked to aniridia with congenital cataract^56–58^. The phenotypes caused by two PAX6 mutations in this study were in accordance with these previous results. Liu *et al*. revealed the PAX6 mRNA level was about 50% lower in patients caused by p.A266fs mutation than in unaffected family members, indicating that this mutation caused nonsense-mediated mRNA decay (NMD)^59^. Since NMD is a common pathogenic mechanism of nonsense and frameshift mutations, we hypothesized that nonsense-mediated decay (NMD) may be the pathogenic mechanism of two PAX6 mutations we identified as well.

In conclusion, our results showed that mutations in the 54 known genes were responsible for about 62.96% of this set of Chinese families with congenital cataracts. And mutations in the crystallin gene were identified in 37.03% of the families. Therefore, we believed that targeted exome sequencing is an efficient method in disease-causing mutation identification.

## Materials and Methods

### Patient Recruitment

The research protocols of this study adhered to the guidelines of the Declaration of Helsinki and were approved by the Medical Ethics Committees of the Second Affiliated Hospital, College of Medicine, Zhejiang University (Hangzhou, China). Appropriate informed consent from each participant was obtained.

Among 27 families, 24 were diagnosed with congenital cataracts, while 3 were diagnosed with aniridia and congenital cataract. 25 families with family history showed autosomal dominant inheritance, and 2 were sporadic patients. Available individuals indicated in Fig. 1 were given complete physical, ophthalmic examinations. One hundred unrelated healthy subjects from the same ethnic background were recruited as controls. Peripheral blood was collected by venipuncture in EDTA-coated Vacutainer tubes (BD, New Jersey, USA) and stored at −20 °C.

### DNA Extraction and Next Generation Sequencing

Genomic DNA of 27 probands was isolated from the 2 ml peripheral blood samples using QIAamp DNA Blood kits (Qiagen, Hilden, Germany). Then the purity and quantity of DNA samples were measured by the NanoDrop 2000 spectrophotometer (Thermo Fisher Scientific, Inc., Waltham, Massachusetts). Genomic DNA was shearing by CovarisTM system. Then sample preparation by following the manufacturer’s standard procedure using Truseq DNA Sample preparation Kit (Illumina, Inc, San Diego, CA).

The coding exons, flanking regions and promotor regions of 54 genes related to inherited cataracts were selected and captured using a SureSelect Target Enrichment Kit (Agilent technologies, Inc, USA). The kit included 5,721 probes and could enrich about 551 exons and cover about 94.7% targeted regions. The enrichment libraries were sequenced on Illumina HiSeq2000 Sequencer (Illumina, Inc, San Diego, CA); the average sequencing depth was 500-fold.

### Bioinformatics Analysis

The low quality reads and adaptor sequences were filtered out with the FASTX program. Picard program was used to remove the PCR duplicates. After high-quality reads were retrieved, the clean data were aligned using BWA program according to human genome parameters (hg19). Subsequently, we determined SNPs using the SOAPsnp program, realigned the reads with BWA, and detected the deletions or insertion (InDels) with the GATK software. After SNPs are identified, we use ANNOVAR to do annotation and classification. Finally, all nonsynonymous variants were evaluated by three algorithms, SIFT (http://sift.jcvi.org/), PolyPhen-2 (http://genetics.bwh.harvard.edu/pph2/), Mutation Tester (http://www.mutationtaster.org/).

### Expanded Validation

DNA samples of probands were taken for further Sanger sequencing, to confirm the potential pathogenic variants detected by exome sequencing. Polymerase chain reaction (PCR) was performed in a 20 μl reaction system using the primer pairs previously published^60^ or designed by Primer Premier 6.0 (Table S3). PCR products were isolated using electrophoresis on 3% agarose gels and sequenced using the BigDye Terminator Cycle sequencing kit V 3.1 (ABI–Applied Biosystems; Sangon Co, China) on an ABI PRISM 3730 Sequence Analyzer (ABI). Sequencing results were analyzed using Chromas 2.3.0 and compared with sequences from NCBI human genome database. Confirmed variants were further sequenced in the all available family members and 100 control individuals.

## Electronic supplementary material

All the supplementary materials
