# Supplementary material for: Targeted Exome Sequencing of Congenital Cataracts Related Genes: Broadening the Mutation Spectrum and Genotype–Phenotype Correlations in 27 Chinese Han Families
Source: Sci Rep. 2017 Apr 27;7:1219. doi: 10.1038/s41598-017-01182-9 (PMC5430819; doi:10.1038/s41598-017-01182-9)
Supplement: Supplementary file 1 — All the supplementary materials [file 41598_2017_1182_MOESM1_ESM.pdf]

# Targeted Exome Sequencing of Congenital Cataracts Related Genes: Broadening the Mutation Spectrum and Genotype–Phenotype Correlations in 27 Chinese Han Families

Yi Zhai[1,2+], Jinyu Li[1,2+], Wangshu Yu[3], Sha Zhu[1,2], Yinhui Yu[1,2], Menghan Wu[1,2], Guizhen Sun[4], Xiaohua Gong[5], Ke Yao[1,2*]

1. Eye Center, Second Affiliated Hospital of Medical College, Zhejiang University, Hangzhou, Zhejiang, China
2. Key Laboratory of Ophthalmology of Zhejiang Province, China
3. The 117th hospital of PLA, Hangzhou, China
4. Naval Convalescent Zone of Hangzhou Sanatorium, Nanjing Military Command, CPLA, Hangzhou, China
5. School of Optometry and Vision Science Program, University of California, Berkeley, California, United States of America

*corresponding. xlren@zju.edu.cn

+these authors contributed equally to this work

# Supplementary Information

| Gene | Inheritance | Genomic DNA | mRNA | Protein |
|---|---|---|---|---|
| EPHA2 | AD/AR | NC_000001.10 | NM_004431.3 | NP_004422.2 |
| FOXE3 | AD/AR | NC_000001.10 | NM_012186.2 | NP_036318.1 |
| GJA8 | AD/AR | NC_000001.10 | NM_005267.4 | NP_005258.2 |
| CRYGD | AD | NC_000002.11 | NM_006891.3 | NP_008822.2 |
| CRYGC | AD | NC_000002.11 | NM_020989.3 | NP_066269.1 |
| CRYGB | AD | NC_000002.12 | NM_005210.3 | NP_005201.2 |
| CRYGA | N/A | NC_000002.12 | NM_014617.3 | NP_055432.2 |
| CRYBA2 | AD | NC_000002.12 | NM_057093.1 | NP_476434.1 |
| KCNJ13 | AD | NC_000002.12 | NM_002242.4 | NP_002233.2 |
| FYCO1 | AR | NC_000003.11 | NM_024513.3 | NP_078789.2 |
| BFSP2 | AD/AR | NC_000003.11 | NM_003571.2 | NP_003562.1 |
| CRYGS | AD | NC_000003.11 | NM_017541.2 | NP_060011.1 |
| EZR | N/A | NC_000006.12 | NM_003379.4 | NP_003370.2 |
| GCNT2 | AR | NC_000006.11 | NM_001491.2 | NP_001482.1 |
| EYA1 | AD | NC_000008.10 | NM_000503.4 | NP_000494.2 |
| TDRD7 | AR | NC_000009.11 | NM_014290.2 | NP_055105.2 |
| SLC16A12 | AD | NC_000010.10 | NM_213606.3 | NP_998771.3 |
| PITX3 | AD/AR | NC_000010.10 | NM_005029.3 | NP_005020.1 |
| VIM | AD | NC_000010.10 | NM_003380.3 | NP_003371.2 |
| PAX6 | AD | NC_000011.10 | NM_001258463.1 | NP_001245392.1 |
| CRYAB | AD | NC_000011.9 | NM_001885.1 | NP_001876.1 |
| MIP | AD | NC_000012.11 | NM_012064.3 | NP_036196.1 |
| GJA3 | AD | NC_000013.10 | NM_021954.3 | NP_068773.2 |
| VSX2 | AR | NC_000014.9 | NM_182894.2 | NP_878314.1 |
| MIR184 | AD | NC_000015.10 | N/A | N/A |
| HSF4 | AD/AR | NC_000016.9 | NM_001040667.2 | NP_001035757.1 |
| MAF | AD | NC_000016.9 | NM_005360.4 | NP_005351.2 |
| TMEM114 | AD | NC_000016.9 | NM_001146336.1 | NP_001139808.1 |
| CRYBA1 | AD | NC_000017.10 | NM_005208.4 | NP_005199.2 |
| FTL | AD | NC_000019.9 | NM_000146.3 | NP_000137.2 |
| LIM2 | AR | NC_000019.9 | NM_030657.3 | NP_085915.2 |
| CHMP4B | AD | NC_000020.10 | NM_176812.4 | NP_789782.1 |
| BFSP1 | AR | NC_000020.10 | NM_001278607.1 | NP_001265536.1 |
| CRYAA | AD/AR | NC_000021.8 | NM_000394.2 | NP_000385.1 |
| CRYBB2 | AD | NC_000022.10 | NM_000496.2 | NP_000487.1 |
| CRYBB3 | AD/AR | NC_000022.10 | NM_000496.2 | NP_000487.1 |
| CRYBB1 | AD/AR | NC_000022.10 | NM_001887.3 | NP_001878.1 |
| CRYBA4 | AD | NC_000022.10 | NM_001886.2 | NP_001877.1 |
| NHS | XL | NC_000023.10 | NM_198270.2 | NP_938011.1 |
| TGFB2 | N/A | NC_000001.11 | NM_001135599.2 | NP_001129071.1 |

| | | | | |
|---|---|---|---|---|
| CYP27A1 | AR | NC_000002.12 | NM_000784.3 | NP_000775.1 |
| SIL1 | AR | NC_000005.10 | NM_001037633.1 | NP_001032722.1 |
| GALT | AR | NC_000009.11 | NM_000155.3 | NP_000146.2 |
| FAM126A | AR | NC_000007.14 | NM_032581.3 | NP_115970.2 |
| WT1 | N/A | NC_000011.10 | NM_000378.4 | NP_000369.3 |
| COL2A1 | AD | NC_000012.12 | NM_001844.4 | NP_001835.3 |
| GFER | AR | NC_000016.10 | NM_005262.2 | NP_005253.3 |
| GALK1 | AR | NC_000017.10 | NM_000154.1 | NP_000145.1 |
| CTDP1 | AR | NC_000018.10 | NM_004715.4 | NP_004706.3 |
| DNM2 | N/A | NC_000019.10 | NM_001005360.2 | NP_001005360.1 |
| DMPK | AD | NC_000019.10 | NM_001081563.2 | NP_001075032.1 |
| ERCC1 | N/A | NC_000019.10 | NM_001983.3 | NP_001974.1 |
| MYH9 | AD | NC_000022.11 | NM_002473.5 | NP_002464.1 |
| OCRL1 | N/A | NC_000023.11 | NM_000276.3 | NP_000267.2 |

Note: AD = autosomal dominant; AR = autosomal recessive; XL = X-linked.

**Table S1. 54 genes related to inherited cataracts**

| SNV ID | Gene | Family ID | mRNA | cDNA | Amino acid |
|---|---|---|---|---|---|
| SNV01 | CRYBA4 | 1 | NM_001886 | c.26C>T | p.A9V |
| SNV02 | CRYGS | 4 | NM_017541 | c.53G>A | p.G18D |
| SNV03 | CRYAA | 4 | NM_000394 | c.312+4A>G | |
| SNV04 | MYH9 | 5 | NM_002473 | c.1270C>T | p.R424W |
| SNV05 | CRYBA1 | 5 | NM_005208 | c.269_271del | p.90_91del |
| SNV06 | HSF4 | 6 | NM_001538 | c.-497-8C>G | |
| SNV07 | CRYGS | 7 | NM_017541 | c.224G>T | p.G75V |
| SNV08 | MYH9 | 8 | NM_002473 | c.3754G>A | p.A1252T |
| SNV09 | HSF4 | 9 | NM_001040667 | c.347G>A | p.R116H |
| SNV10 | CRYBA1 | 9 | NM_005208 | c.607C>T | p.Q203X |
| SNV11 | EZR | 10 | NM_003379 | c.1597-7insTAAT | |
| SNV12 | EZR | 10 | NM_003379 | c.1439C>T | p.P480L |
| SNV13 | MYH9 | 13 | NM_00247 | c.2900T>C | p.V967A |
| SNV14 | VIM | 14 | NM_003380 | c.623A>G | p.Q208R |
| SNV15 | MIP | 15 | NM_012064 | c.607-1G>A | |
| SNV16 | CRYBB2 | 16 | NM_000496 | c.463C>T | p.Q155X |
| SNV17 | CRYBB2 | 17 | NM_000496 | c.452G>A | p.W151X |
| SNV18 | GJA3 | 17 | NM_021954 | c.304A>C | p.M102L |
| SNV19 | FYCO1 | 18 | NM_024513 | c.3215C>T | p.A1072V |
| SNV20 | CRYBA2 | 18 | NM_057093 | c.343A>G | p.N115D |
| SNV21 | BFSP1 | 19 | NM_001278607.1 | c.625+3A>G | |
| SNV22 | GJA8 | 20 | NM_005267 | c.98G>A | p.R33Q |
| SNV23 | OCRL | 21 | NM_001587 | c.430G>T | p.V144F |
| SNV24 | CRYGD | 22 | NM_006891 | c.70C>A | p.P24T |
| SNV25 | EPHA2 | 22 | NM_004431 | c.1339C>T | p.R447C |
| SNV26 | PAX6 | 24 | NM_001258462 | c.795delA | p.E265fs |
| SNV27 | CRYGD | 26 | NM_006891 | c.43C>A | p.R15S |
| SNV28 | PAX6 | 27 | NM_001258462 | c.342G>A | p.W114X |
| SNV29 | LIM2 | 28 | NM_030657 | c.514C>T | p.R172C |
| SNV30 | SIL1 | 28 | NM_022464 | c.1115G>A | p.G372D |

**Table S2. Potential pathogenic mutations in 27 Chinese families with congenital cataract.**

| SNV ID | Gene | Primers | |
|---|---|---|---|
| | | F | R |
| SNV01 | CRYBA4 | 5'-TGGAAGATGGCATTGGTGACA-3' | 5'-GGATTCATGGGGACCTGAACC-3' |
| SNV02 | CRYGS | 5'-AATTAAGCCACCCAGCTCCT-3' | 5'-AAGCAAGAGAAAGCGGACAG-3' |
| SNV03 | CRYAA | 5'-CACCTGACCATAGCCAAACAAC-3' | 5'-TCTCCCAGGGTTGAAGGCA-3' |
| SNV04 | MYH9 | 5'-AGATCTCGAAGCCGGCAATGT-3' | 5'-TTGGCCAAGGCGACCTATGAG-3' |
| SNV05 | CRYBA1 | 5'-GCCTTCTCCCCAAGGCCATA-3' | 5'-CCCTCATATGCACGGAAGTGG-3' |
| SNV06 | HSF4 | 5'-CCCGCGCCTGCGCACCTATAC-3' | 5'-GTACCCCGCGCGTTCCCTGTC-3' |
| SNV07 | CRYGS | 5'-TCTTCAAAACACTCAAGAGGCAGAGA-3' | 5'-TGTCAGAAAGCAATGTTGTAAGGTGA-3' |
| SNV08 | MYH9 | 5'-TCCTTGGTGAGCTTGCTGGAC-3' | 5'-GACCAGCCAGCCTTGTGTCAG-3' |
| SNV09 | HSF4 | 5'-GGCACCGCTCACCCTCCTGGT-3' | 5'-CGCAGGAGCAAGGCAGGCAGT-3' |
| SNV10 | CRYBA1 | 5'-ATCTCCCCCAGACATGCCTCT-3' | 5'-TGTGGATTTCAGCAGCTAACATTTG-3' |
| SNV11 | EZR | 5'-TCTAGTGAGGGCATCCG-3' | 5'-TTCCGTAATTCAATCAGTCC-3' |
| SNV12 | EZR | 5'-CAGGACAGAGGGAGGTGA-3' | 5'-ACATTAAGCAGCATTGGTCTA-3' |
| SNV13 | MYH9 | 5'-CTCCAGGGAGCACTTGCAGTT-3' | 5'-TCTGCGTCCTGCCTGTTTCAT-3' |
| SNV14 | VIM | 5'-GGAGCGAGACAAAGGGATGGT-3' | 5'-TGTGGTCTTCCCGCCACTAGA-3' |
| SNV15 | MIP | 5'-GCTTCTACAGGGCCTGGGTGT-3' | 5'-TTCCACTAAGGTGGCTGGAAAAA-3' |
| SNV16 | CRYBB2 | 5'-CACCCTTCCTAGTGGCTTATGGATG-3' | 5'-GGACTGCACCTGGGGGTGA-3' |
| SNV17 | CRYBB2 | 5'-AGTGGCTTATGGATGCTCTATCTCT-3' | 5'-AAGTCGCTGCTGTCCTTGTAGT-3' |
| SNV18 | GJA3 | 5'-GCCCACCTCGAACAGCGTCTTGA-3' | 5'-GGAGGACGTGTGGGGCGATGA-3' |
| SNV19 | FYCO1 | 5'-GTGGCATGCTTCCAGCAGAA-3' | 5'-TGGCACACCAGCCACTTTATG-3' |
| SNV20 | CRYBA2 | 5'-TTTCCTCCACCCCCTCATGTT-3' | 5'-TGCCTGTGGGTCCTAGACAGC-3' |
| SNV21 | BFSP1 | 5'-AGCCACTCTGCACTGGACACC-3' | 5'-TTTCTGCCAGGCTGACCTCTG-3' |
| SNV22 | GJA8 | 5'-CAGATATTGACTCAGGGTTG-3' | 5'-GATGATGTGGCAGATGTAGG-3' |
| SNV23 | OCRL | 5'-CAGTGTCATGGCAAGCACAGC-3' | 5'-ACTGGTCAGTGGGCCTGGACT-3' |
| SNV24 | CRYGD | 5'-TGCTTGAAACCATCCAGTGAGTG-3' | 5'-GCGACCAAAACCCACAACAAG-3' |
| SNV25 | EPHA2 | 5'-TGCCTGCTCGTAGGCAGCTT-3' | 5'-CTCTGCTGTGCTGCCTTGGG-3' |
| SNV26 | PAX6 | 5'-TCTCCATTTGGCCCTTCGATTA-3' | 5'-GGGAGGTGGGAACCAGTTTGA-3' |
| SNV27 | CRYGD | 5'-CCTCGCCTTGTCCCGC-3' | 5'-TTAACTTTTGCTTGAAACCATCCA-3' |
| SNV28 | PAX6 | 5'-TTCCTCTCTGTTCCCCCAGGT-3' | 5'-ATGCAGGTGTCCAACGGATGT-3' |
| SNV29 | LIM2 | 5'-GTGGGACTCATGTGGGACACC-3' | 5'-CCCAAACCTATCCCCCAATCA-3' |
| SNV30 | SIL1 | 5'-AGCCAGCACTGCCAAGATGTC-3' | 5'-CCTCGTGCCTTCCTGTCTGAA-3' |

**Table S3. Primers used to amplify and sequence the variants regions in this study.**

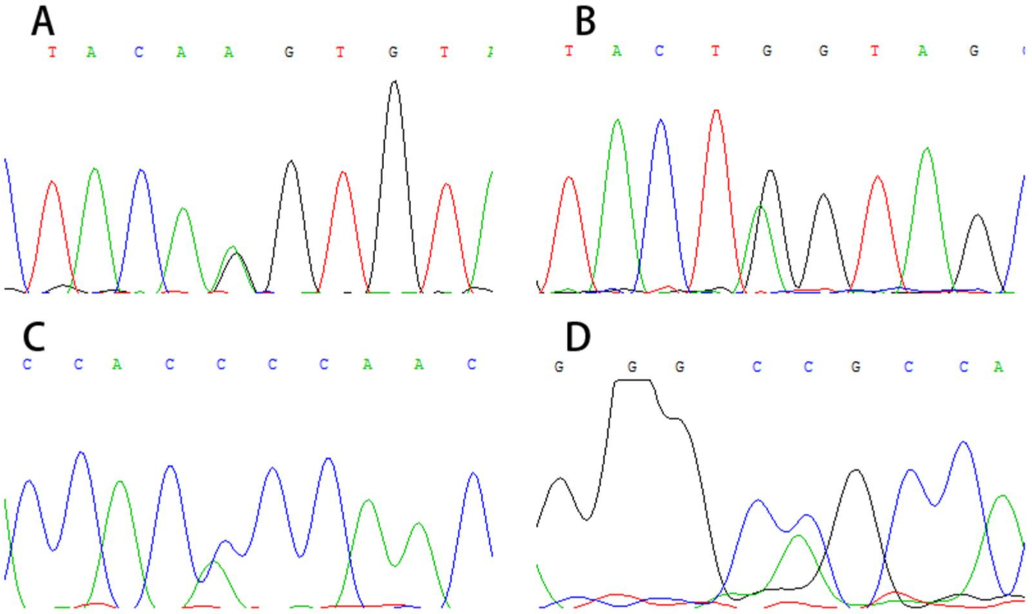


**Figure S1. Sequencing results of four recurrent disease-causing mutations.** (A) Forward sequencing showed c.607-1G>A mutation of MIP gene in patients from family 15. (B) Reverse sequencing showed c.463C>T mutation of CRYBB2 gene in patients from family 16. (C) Forward sequencing showed c.70C>A mutation of CRYGD gene in patients from family 22. (D) Forward sequencing showed c.43C>A mutation of CRYGD gene in patients from family 26.

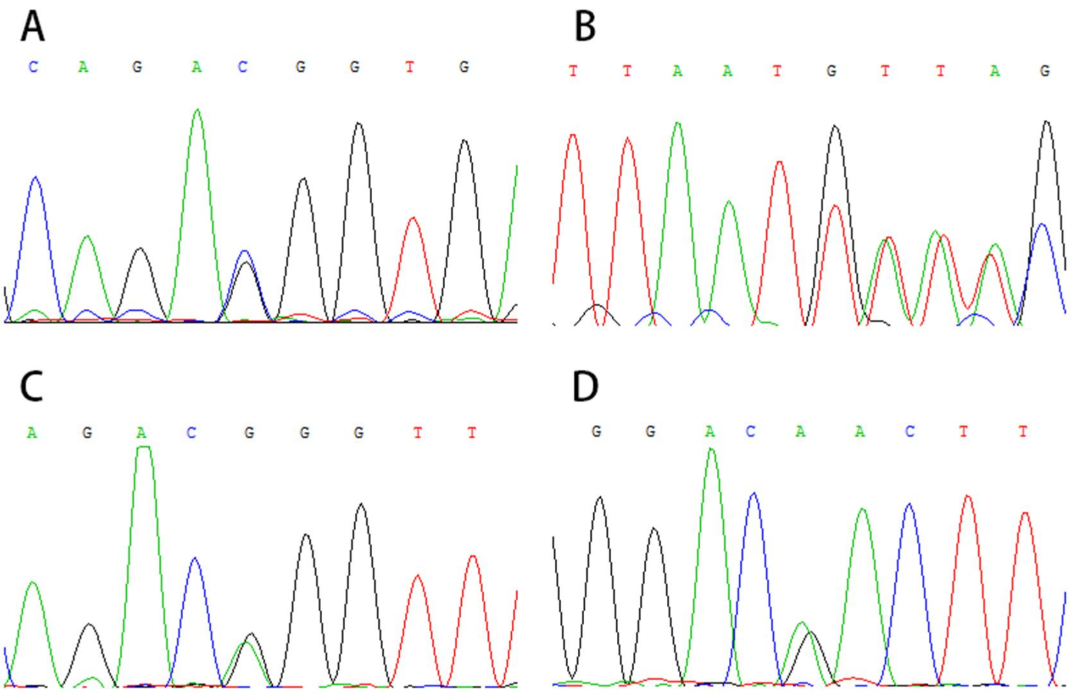


**Figure S2. Sequencing results of four four pathogenic mutations could not be strongly associated with congenital cataracts.** (A) Forward sequencing showed c.-497-8C>G mutation of HSF4 gene in patients from family 6. (B) Forward sequencing showed c.1597-7insTAAT mutation

of EZR gene in patients from family 10. (C) Forward sequencing showed c.623A>G mutation of VIM gene in patients from family 14. (D) Forward sequencing showed c.343A>G mutation of CRYBA2 gene in patients from family 18.
